# Near-Full-Length Genome Sequences Representing an Event of Zooanthroponotic Transmission of SARS-CoV-2 Lineage B.1.189 in Mexico during 2020

**DOI:** 10.1128/mra.00497-22

**Published:** 2022-07-19

**Authors:** Roberto Navarro-Lopez, Mario Solis-Hernandez, Marisol K. Rocha-Martinez, Samantha Eberl, Ninnet Gomez-Romero, Lauro Velazquez-Salinas, J. Guillermo Estrada-Franco

**Affiliations:** a Comision Mexico–Estados Unidos para la Prevencion de la Fiebre Aftosa y Otras Enfermedades Exoticas de los Animales (CPA), Mexico City, Mexico; b Centro Nacional de Servicios de Constatacion en Salud Animal (CENAPA), Morelos, Mexico; c Department of Psychological Science, Central Connecticut State University, New Britain, Connecticut, USA; d College of Veterinary Medicine, Kansas State University, Manhattan, Kansas, USA; e Centro de Biotecnologia Genomica del Instituto Politecnico Nacional, Reynosa, Tamaulipas, Mexico; Queens College CUNY

## Abstract

Here, we report three near-full-length genome sequences of severe acute respiratory syndrome coronavirus 2 (SARS-CoV-2) obtained in Mexico City, Mexico, during the pandemic of coronavirus disease 19 (COVID-19) in 2020, representing a zooanthroponotic transmission event between humans and a dog. All three genomes belong to the B.1.189 lineage based on the pangolin classification.

## ANNOUNCEMENT

Considered the biggest sanitary event of the century, the coronavirus disease (COVID-19) pandemic is caused by the severe acute respiratory syndrome coronavirus 2 (SARS-CoV-2), a member of the *Coronaviridae* family within the *Betacoronavirus* genus. To date (8 May 2022), the cases and deaths produced by this virus have been 513,955,910 and 6,249,700, respectively. Mexico has been one of the countries with the highest number of deaths (324,334) during this pandemic (https://covid19.who.int/), representing 5.18% of the total mortality worldwide.

The remarkable genome plasticity displayed by SARS-CoV-2 (1) leads to the divergence of multiple phylogenetic clades and the consequent emergence of different viral variants of concern (2). Therefore, the control of this pandemic has represented a major challenge (3, 4).

Recent reports documented the zooanthroponotic spillover of variants of concern like Delta (cats, dogs, pumas, lions, and hamsters) and Omicron (white-tailed deer) in wild and domestic animals (5, 6). Thus, documented infections produced by human-to-animal transmission are increasing (7, 8).

**FIG 1:**
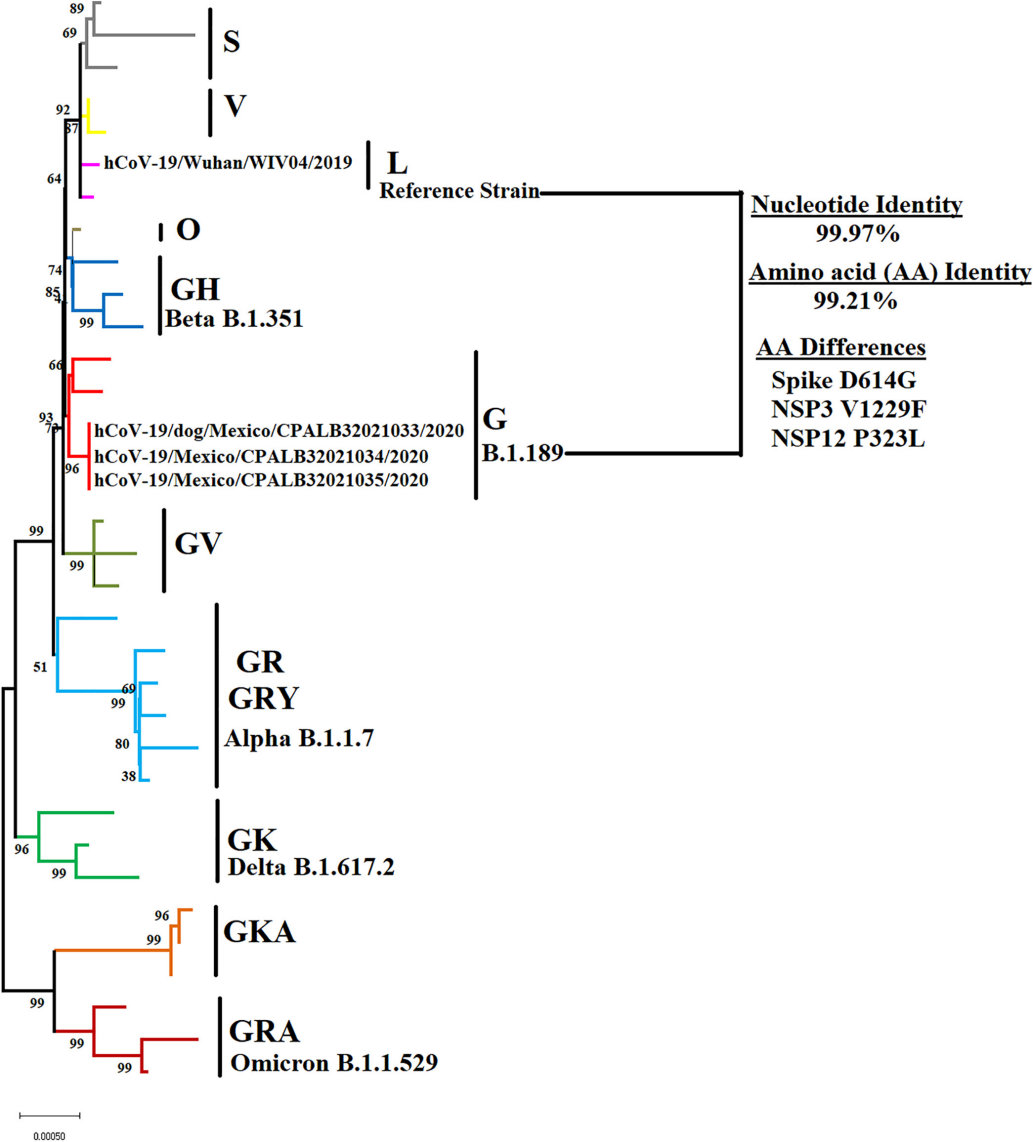
Phylogenetic tree of SARS-CoV-2 from a zooanthroponotic spillover case in Mexico City during 2020. The zooanthroponotic event described here involved two humans and a dog (Canis lupus familiaris) living in a household. The phylogenetic analysis was conducted by maximum likelihood and the general time reversible model, showing the genetic relationship of sequences reported in this study with different divergent clades of SARS-CoV-2 (GISAID classification) and multiple variants described during the pandemic. In addition, results of the comparison between sequences and the reference strain sequence are shown. The analysis involved a total of 31 representative sequences of different clades obtained from the GISAID database (9).

Here, we report three near-full-length genome sequences of SARS-CoV-2 strains obtained from nasopharyngeal swab specimens recovered during a zooanthroponotic spillover event between humans and a dog in Mexico City, Mexico, in 2020. All sequences were classified as part of the pangolin lineage B.1.189 (Fig. 1). Interestingly, no changes were observed in the consensus sequence obtained from the dog, showing the apparent genetic stability of this lineage after infection in different species.

Viral isolation was performed in Vero cells (ATCC C1008). Subsequently, RNA from the three viral isolates was extracted using the high pure viral RNA kit (Roche), following the manufacturer’s protocol. Next-generation sequencing (NGS) of amplicons was conducted to obtain the SARS-CoV-2 sequences reported in this announcement. For this purpose, a set of 15 primers were developed to cover the genome of SARS-CoV-2 (Table 1). Reverse transcriptase PCR (RT-PCR) reactions were conducted using the SuperScript III one-step RT-PCR system with Platinum *Taq* DNA polymerase kit, following the manufacturer’s instructions. Libraries were prepared using the Nextera XT DNA library preparation kit following the manufacturer’s protocol. Sequencing and analyses were conducted on the MiSeq system (Illumina). Raw data of samples identified as hCoV-19/dog/Mexico/CPALB32021033/2020, hCoV-19/Mexico/CPALB32021034/2020, and hCoV-19/Mexico/CPALB32021035/2020 consisting of 3,348,413, 8,248,286, and 9,425,298 reads, respectively, with an average read length of 200 bp were analyzed. All analyses were performed in CLC Genomics Workbench v11.0. The paired reads were quality trimmed using default parameters. Reads were then mapped to the reference strain sequence (GenBank accession number NC045512.2). Consensus sequences were obtained using default parameters and annotated based on a comparison with the reference strain. All work conducted in humans and animals was approved by bioethics committee Escuela Nacional de Medicina y Homeopatía (ENMH) number CBE/006/2020 on the project “Zoonosis Virales Emergentes en Tiempos de Circulación de COVID-19 en México.”

**TABLE 1:** Sequencing considerations^*a*^

Amplicon no.	Primer ID^*b*^	Primer sequence (5′–3′)	Location in reference sequence	Size (bp)	Annealing temp (°C)
1	1FCOVID	GCC TTC CCA GGT AAC AAA CCA ACC	15–1931	1,916	58
	2RCOVID	GAG CAG TTT CAA GAG TGC GGG AG			
2	3FCOVID	GCA TTT GCA TCA GAG GCT GCT CG	1868–4148	2,280	56
	4RCOVID	CAC CCT CTT GAA CAA CAT CAC CCA C			
3	5FCOVID	GGC AAT CTT CAT CCA GAT TCT GCC	4046–6371	2,325	58
	6RCOVID	TTC CCT GCG CGT CCT CTG ACT TC			
4	7FCOVID	GTA CCA AAC CAA CCA TAT CCA AAC GC	6008–8372	2,364	56
	8RCOVID	CCT GCG CAT TAA TAT GAC GCG CAC			
5	9FCOVID	CAG CAG CTC GGC AAG GGT TTG TTG	8169–10209	2,040	58
	10RCOVID	GGG TTA AGC ATG TCT TCA GAG GTG C			
6	11FCOVID	CCA CAA ACC TCT ATC ACC TCA GCT G	10022–12261	2,239	56
	12RCOVID	CGT TGC ATG GCT GCA TCA CG			
7	13FCOVID	GGG CAA CCT TAC AAG CTA TAG CC	12078–14333	2,255	55
	14RCOVID	CAA TTT GGG TGG TAT GTC TGA TCC C			
8	15FCOVID	CTG CAG AGT CAC ATG TTG ACA CTG	14195–16411	2,216	55
	16RCOVID	CTG TGA CAT CAC AAC CTG GAG C			
9	17FCOVID	CAC ACC GCA TAC AGT CTT ACA GGC	16215–18466	2,251	58
	18RCOVID	CAG GCG GTG GTT TAG CAC TAA C			
10	19FCOVID	CGA TGT CGA GGG GTG TCA TGC TAC	18306–20099	1,793	57
	20RCOVID	GCT TGT TTG GGA CCT ACA GAT GG			
11	21FCOVID	GGG TGT GGA CAT TGC TGC TAA TAC	19845–22446	2,601	56
	22RCOVID	GGG TCA AGT GCA CAG TCT ACA GC			
12	23FCOVID	GTT GGA CAG CTG GTG CTG CA	22332–24239	1,907	58
	24RCOVID	CAG CAC CTG CAC CAA AGG TCC AAC			
13	25FCOVID	GCC ACC TTT GCT CAC AGA TGA AAT G	24145–26353	2,208	55
	26RCOVID	GCG CAG TAA GGA TGG CTA GTG			
14	27FCOVID	CGA CGA CGA CTA CTA GCG TGC	26192–28375	2,183	60
	28RCOVID	CCC ACT GCG TTC TCC ATT CTG G			
15	29FCOVID	GCA CCC CGC ATT ACG TTT GGT G	28307–29798	1,491	60
	30RCOVID	CTT CCA TAT AGG CAG CTC TCC CTA GC			

a Description of multiple sets of primers developed in this study to conduct the NGS amplicon sequencing described in this study. The location of the primers corresponds to the nucleotide positions in the reference sequence of SARS-CoV-2 under the accession no. NC045512.2.

b ID, identification.

The information from these events is useful for defining the potential role of dogs as reservoirs or intermediate hosts of SARS-CoV-2. In addition, future studies may help evaluate the possible differences in the transmission in animal species among SARS-CoV-2 lineages.

### Data availability.

Sequences are available in the Global Initiative on Sharing All Influenza Data (GISAID) database under the following accession numbers: EPI_ISL_11991713 (hCoV-19/dog/Mexico/CPALB32021033/2020), EPI_ISL_11988443 (hCoV-19/Mexico/CPALB32021034/2020), and EPI_ISL_11988444 (hCoV-19/Mexico/CPALB32021035/2020). The raw sequencing data of this project are available in the NCBI Sequence Read Archive (SRA) under the BioProject number PRJNA827138.
